# Synthesis of Known and New Host Plant Records of the Fijian Ginger Weevil, *Elytroteinus geophilus* (Lucas) (Coleoptera, Curculionidae, Cryptorhynchinae) Suggests a Preference for Starch-storing Plant Organs

**DOI:** 10.3390/insects10080229

**Published:** 2019-07-31

**Authors:** Ryan J. Whitehouse, M. Lourdes Chamorro

**Affiliations:** 1 Mississippi Entomological Museum, Mississippi State University, Mississippi State, MS 39762, USA; 2 Systematic Entomology Laboratory, Agricultural Research Service, United States Department of Agriculture, c/o National Museum of Natural History, Smithsonian Institution, Washington, DC 20013, USA

**Keywords:** quarantine pest, *Elytroteinus subtruncatus*, Hawaii, *Crinum* sp.

## Abstract

*Elytroteinus geophilus* (Lucas) is a polyphagous weevil that is widespread in the South Pacific islands and is known to cause damage to various crops with large nutrient storage structures such as kava, ginger, yellow passion fruit, yams and sweet potatoes. More significantly, *E. geophilus*, the Fijian Ginger Weevil, has been linked, along with two wound invading fungal pathogens, to a passion fruit collar rot in Samoa. This species is considered a high-risk insect pest and it is included in the USDA’s prioritized offshore pest list. We report on new plant hosts and behavior of this weevil. The first new host record resulted from interception of this weevil in bulbs of Tropical Spider Lily (*Crinum* sp.) in Alabama. This interception initiated an examination of museum specimens and the literature that resulted in a second previously unreported host record (vanilla (Orchidaceae)) and a new behavioral trait for this weevil: the use of plant fibers to spin a cocoon for pupation. A synthesis of known host plants records is reported here and suggests a preference by this weevil of starch-storing plant organs. A distribution map and a differential diagnosis of the species is also provided.

## 1. Introduction

The Fijian Ginger Weevil, *Elytroteinus geophilus* (Lucas, 1861), is a polyphagous weevil known to cause severe damage to mostly roots (particularly near the base of the stem), corms, and tubers of a wide range of host plants (Table 1) in many different orders and families. Most reports in the literature are from dead or dying plants or stored products rather than living and healthy plants [1,2,3,4]. More significantly, the Fijian Ginger Weevil has been linked, along with two wound invading fungal pathogens (*Lasiodiplodia theobromae* (Pat.) Griffon & Maubl. and *Haematonectria haematococca* (Berk. & Broome) Samuels & Nirenberg (asexual name *Fusarium solani* (Mart.) Sacc.) causing passion fruit collar rot in Samoa [5,6]. 

*Elytroteinus geophilus* is considered to be a significant threat to U.S. agriculture, as listed in the U.S. Department of Agriculture’s prioritized offshore pest list [7]. In 1995 and 1997, this weevil was intercepted at the Keahole International Airport in Hawaii in sweet potatoes in passenger baggage (Convolvulaceae) [1]. This led to the listing of the Fijian Ginger Weevil as a federal quarantine pest for sweet potato in the United States. However, Follett et al. [1] were not able to find this weevil in commercial sweet potato fields in Hawaii or in stored sweet potatoes. 

**Figure 1:**
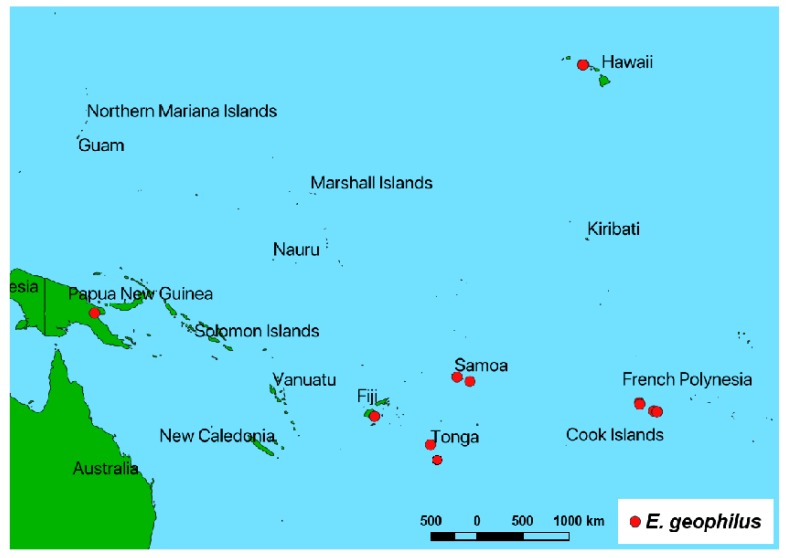
Known distribution of *E. geophilus*.

*Elytroteinus geophilus* is native to the Australian-Malayan area and has spread to multiple islands through human activity [10,13] (Figure 1). The Fijian Ginger Weevil is commonly treated in the literature, as recently as 2012, by its junior synonym, *Elytroteinus subtruncatus* (Fairmaire). This synonymy by Kuschel [14] has at times been overlooked [7]. Two additional species are currently included in *Elytroteinus* Marshall, 1920: *Elytroteinus recticollis* Zimmerman, 1938 from Solomon Islands and *Elytroteinus vitiensis* Kuschel, 2008 from Fiji. The type specimen of *E. recticollis* has been digitized by the Museum of Comparative Zoology, Harvard University (MCZ) accessible through their website.

*Elytroteinus geophilus* was first discovered in Fiji in 1881 [2]. It was first reported in Hawaii in 1918, feeding on the rhizomes of common white ginger, *Hedychium coronarium* (Zingiberaceae) [15]. 

Currently, little is known about the natural history of this weevil. In kava, (Piperaceae) the females will make oviposition holes in the stems where they lay their eggs [11]. The eggs will then hatch and larvae tunnel inside the stems, weakening the plant and making it susceptible to bacteria or fungi [11]. When found in lemons (Rutaceae) on Cook Island, the larvae were found near the base of the stalk, working their way through the peel and attacking the core [2]. Only one larva was found per fruit and pupation occurred within the fruit [2]. This species has also been reported as a stem borer in begonias (Begoniaceae) and is found in the main stems often near the base [9].

On 1 April 2019 in Scottsboro, Alabama, bulbs of Tropical Spider Lily, *Crinum* sp. (Amaryllidaceae), originally purchased in Hawaii, were discovered to be infested with weevils and sent to the Mississippi Entomological Museum (MEM) and forwarded to the United States National Museum (USNM) for identification. We report our findings and include occurrence information, a list of known host records, a distribution map, and a differential diagnosis of the species.

## 2. Materials and Methods

**Figure 2:**
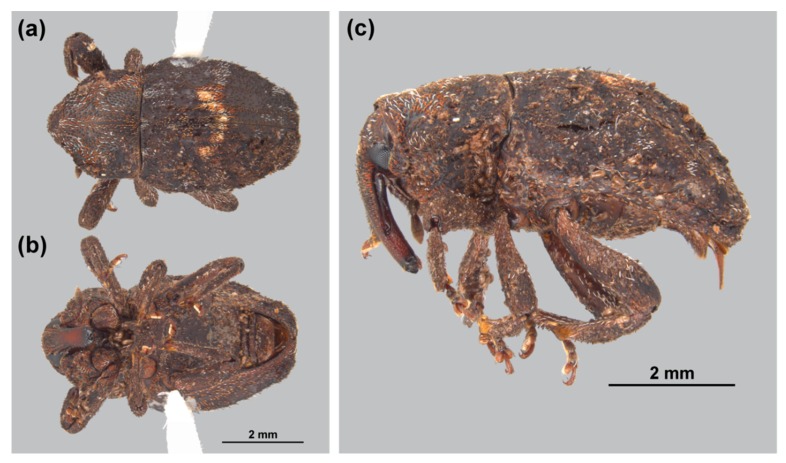
Habitus of *Elytroteinus geophilus*: (**a**) dorsal; (**b**) ventral; and (**c**) lateral views.

The bulbs sent to the MEM were carefully dissected to reveal two adult weevils. The specimens were imaged using a Leica DFC 495 digital camera mounted on a Leica Z16 microscope and Leica Application Suite was used to create image stacks (Figure 2). The images were then sent to Lourdes Chamorro at the USNM to help with the identification of the weevils. One specimen is deposited at the MEM and the other at the USNM. The following resources were used to identify this weevil: Zimmerman [16] and authoritatively identified specimens housed in the USNM. 

Records from the Systematic Entomology Laboratory’s Communications & Taxonomic Service Unit (CTSU) internal identification system were queried in 2016 for information on interception records. 

The distribution map was made using QGIS [17] with data from specimens at the USNM and from specimens found in the Symbiota Collections of Arthropods Network (SCAN) database [18]. MycoBank.org [19] was used to check the names of the fungal pathogens. 

## 3. Results

The weevils dissected from *Crinum* sp. were identified as Fijian Ginger Weevils, *Elytroteinus geophilus*. This is the first record of Fijian Ginger Weevil in Amaryllidaceae (*Crinum* sp.). In addition, following examination of the U.S. National Collection., a new host record, Orchidaceae (*Vanilla planifolia* Jacks. ex Andrews) and a new behavioral trait for this weevil: the use of plant fibers to spin a cocoon for pupation are here reported. 

**Figure 3:**
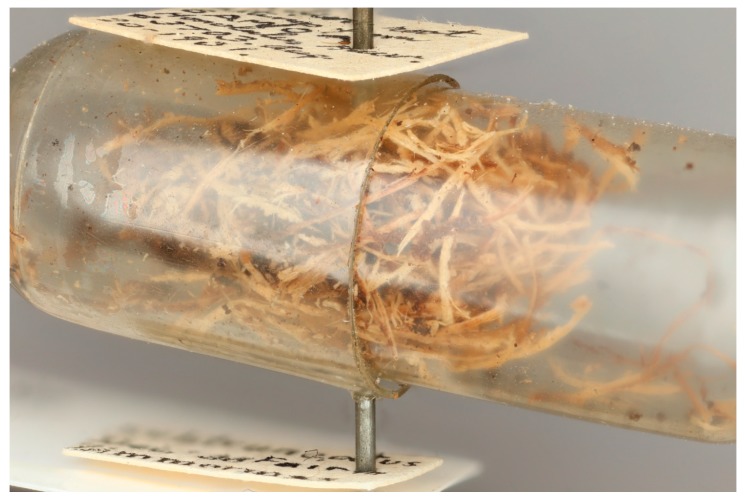
Fibrous cocoon found with specimen of *E. geophilus* that was intercepted in Honolulu, Hawaii on material originating in Samoa.

The USNM weevil collection houses 15 specimens of *E. geophilus*. The earliest record is of two specimens from Tahiti collected in 1916 from *Vanilla planifolia* Jacks. ex Andrews (Orchidaceae). The most recent record is from 1978 of a specimen intercepted in Hawaii from Tonga in yam root (Dioscoreaceae). Three additional specimens are from Tahiti, two collected on *Zingiber* sp. and the third on ginger (*Zingiber officinale* Roscoe). Most specimens in the collection were taken from yam (*Dioscorea*), including two specimens reared in 1918 in San Francisco by the California State Commission of Horticulture from yams originating from Fiji. A specimen intercepted in 1931 in Honolulu, originating from Samoa in A[illegible]a root, was preserved with its fiber cocoon (Figure 3). The use of fibers to spin a cocoon was not a previously reported behavior of this species. In addition to the specimens in the USNM’s weevil collection, at least 11 more specimens of *E. geophilus* have been intercepted between the years 1995–2013 and 18 more occurrence records are reported in SCAN.

The SCAN database has 29 occurrence records of *E. geophilus* (under the junior name *E. subtruncatus*) with 15 from the United States National Museum’s Entomology Collection (USNM), 11 from the University of Hawaii Insect Museum (UHIM), two from the Hawaii Department of Agriculture, Pest Control Branch (HDOA), and one from the Arizona State University Charles W. O’Brien Collection (ASUCOB). All of the specimens from collections besides the United States National Museum’s Entomology Collection were collected in Hawaii between 1918 and 2010.

## 4. Discussion

The risk posed by the Fijian Ginger Weevil has been well recognized by regulatory agencies (USDA prioritized list). The Fijian ginger weevil successfully develops and is commonly encountered in a wide range of plant species, families, and orders. However, the common denominator appears to be the preference by the weevil of the plant’s enlarged nutrient storage structures, such as tubers, bulbs, rootstalks, and seeds. Therefore, the preference of this weevil is not necessarily for a particular evolutionary plant lineage (i.e. Zingiberales or Poales), but for plants with a specific metabolic strategy, ones with large starch-storing plant organs. 

This newly discovered association between the Fijian Ginger Weevil and Tropical Spider Lily and vanilla, represent potential new entry pathway for this weevil into the country regardless of taxonomic status. This species currently has a narrow distribution range, restricted largely to the South Pacific (Figure 1), which makes monitoring for this species relatively manageable. The discovery of the previously unreported behavior of cocoon construction by the preimaginal stage using plants fibers is significant because in some instances, the presence of the weevil in the plant may go unnoticed. The behavioral trait of constructing a fibrous pupal cocoon is also exhibited by most dryophthorine weevils, particularly species associated with monocots [20,21,22]. 

While the identity of this species has been well established, the valid name *E. geophilus*, has been mostly overlooked. When searching SCAN for specimen records, none of the records were identified as *E. geophilus*, instead they were all identified as *E. subtruncatus*. Most of the available literature on this species [1,2,3,4,5,7,9,10,13,15,16] and multiple websites and databases [18,23,24] before the publication of this paper had the name *E. subtruncatus* in use. This is most likely because the synonymy of *E. subtruncatus* with *E. geophilus* was relatively recent and has largely gone unnoticed given that the taxonomic act [14] was published in a difficult to access book chapter. Also, the lack of an updated species checklist or catalog of the weevils of the South Pacific region may also be a contributing factor.

*Elytroteinus* belongs to the cryptorhynchine subtribe Tylodina and as such possesses a bare, cavernous rostral channel that extends beyond the prosternum to the anterior margin of the mesocoxae, lacks a dorsally visible scutellum and elytral humeri, has a short metasternum and a narrow or vestigial metepisternum, and at least the fore femora are toothed, among other characters. *Elytroteinus geophilus* can be identified as a moderately large (6.56–6.64 mm long (anterior portion of head to elytral apex), 6.4–6.48 mm long (pronotum to elytral apex), 3.6 mm wide at widest point (n = 2)), dark brown weevil with brown, reddish-brown, and white, appressed widely spaced scales on the pronotum and elytra. At approximately the midpoint of the elytra, there is a distinctive, W-shaped band of white scales, but this feature can be indistinct in rubbed specimens. In addition to the distinctive scale pattern, this species can be recognized by having the elytra with subtruncate apex, with the declivity starting at midpoint, and inflexed sides; profemora each with a large distinct tooth that is greatly reduced on the meso- and meta-femora; and integument matte and only noticeably shining on the apical half of the rostrum. *Elytroteinus geophilus* can be distinguished from *E. recticollis* by the lack of distinct punctations on the pronotum and by the pronotal sides being curved in the basal two thirds. In addition, the scales of *E. geophilus* are more numerous and longer than in *E. recticollis.*

## 5. Conclusions

Through examination of museum specimens and a new domestic interception record, two new host records were found for *E. geophilus*, Tropical Spider Lily (*Crinum* sp., Amaryllidaceae) and vanilla (*Vanilla planifolia*, Orchidaceae). The behavior of making a fibrous cocoon by this species is also reported for the first time. A synthesis of known and new host plants records is here reported and suggests a preference by this weevil of starch-storing plant organs. These new discoveries have the potential to significantly impact current management strategies aimed at mitigating the spread of this polyphagous high-risk weevil.

## Figures and Tables

**Table 1:** List of host records and plant associations of *E. geophilus*.

Order	Family	Scientific Name	Common Name	Country	Source
Alismatales	Araceae	*Colocasia esculenta*	taro corms	Hawaii	[1]
Asparagales	Amaryllidaceae	*Crinum sp.*	tropical spider lily	Hawaii	USNM, MEM
Asparagales	Asparagaceae	*Coryline fruticosa*	ti cuttings	Hawaii	[8]
Asparagales	Asparagaceae	*Ophiopogon japnonicus*	dwarf mondo roots	Hawaii	[1]
Asparagales	Orchidaceae	*Vanilla planifolia*	vanilla	Tahiti	USNM
Asparagales	Asphodelaceae	*Hemerocallis sp.*	daylily	Hawaii	[4]
Cucurbitales	Begoniaceae	*Begonia*	Begonia	Fiji	[9]
Cycadales	Cycadaceae	*Cycas sp.*	cycad trunks	Hawaii	[4]
Dioscoreales	Dioscoreaceae	*Dioscorea sp.*	yam	Fiji and Tonga	USNM
Fabales	Fabaceae	*Inocarpus fagifer*	Tahitian chestnut fruits	Society Islands	[10]
Laurales	Lauraceae	*Persea americana*	Avocado seeds	Hawaii	[4]
Malpighiales	Passifloraceae	*Passiflora edulis var. flavicarpa*	yellow passion fruit	Samoa	[5]
Marattiales	Marattiaceae	*Marattia douglasii*	Marattia fern	Tahiti	[4]
Piperales	Piperaceae	*Piper methysticum*	kava	Tonga	[11]
Poales	Poaceae	*Saccharum officinarum*	sugarcane	Samoa	[3]
Sapindales	Rutaceae	*Citrus limon*	lemons	Cook Island	[2]
Solanales	Convolvulaceae	*Ipomoea batatas*	sweet potato	Hawaii	[1]
Zingiberales	Strelitziaceae	*Strelitzia reginae*	bird of paradise tubers	not reported	[12]
Zingiberales	Zingiberaceae	*Hedychium coronarium*	common white ginger	Hawaii	[4]
Zingiberales	Zingiberaceae	*Zingiber officinale*	ginger root	Tahiti	[1], USNM

## References

[1] Follett P.A., Alontaga D., Tom R., Weinert E.D., Tsuda D., Kinney K. (2007). Absence of the quarantine pest *Elytroteinus subtruncatus* in east Hawaii sweetpotato fields. Proc. Hawaii Entomol. Soc..

[2] Miller D. (1923). The Fiji lemon-weevil (*Elytroteinus subtruncatus* Frm.). N. Z. J. Agric. Res..

[3] Swezey O.H. (1923). Notes on insect pests in Samoa. Proc. Hawaii Entomol. Soc..

[4] Swezey O.H. (1952). Notes and Exhibitions. Proc. Hawaii Entomol. Soc..

[5] Liebregts W., Sands D., Bourne A. (1989). Population studies and biological control of *Pseudaulacaspis pentagona* (Targioni-Tozzetti) (Hemiptera: Diaspididae) on passion fruit in Western Samoa. Bull. Entomol. Res..

[6] Pacific Pests and Pathogens- Fact Sheets, Ginger Weevil (357). https://apps.lucidcentral.org/ppp/text/web_full/entities/ginger_weevil_357.htm.

[7] The 2012 Prioritized Offshore Pest List. https://www.aphis.usda.gov/plant_health/plant_pest_info/pest_detection/downloads/farmbill/PrioritizedOffshorePestList.pdf.

[8] Ford E.J. (1955). Note and Exhibitions. Proc. Hawaii Entomol. Soc..

[9] Simmonds H.W. (1928). Entomological Notes—*Elytroteinus subtruncatus*, Fairm. Agric. J. Dept. Agric. Fiji.

[10] Zimmerman E.C. (1936). Cryptorhynchinae of the Society Islands. Bishop Mus. Occas. Pap..

[11] Davis R.I., Brown J.F. (1999). Kava (Piper methysticum) in the South Pacific: Its Importance, Methods of Cultivation, Cultivars, Diseases and Pests.

[12] Rosa J. (1959). Notes and Exhibitions. Proc. Hawaii Entomol. Soc..

[13] Zimmerman E.C. (1938). A second species of *Elytroteinus* (Coleoptera, Curculionidae). Proc. Hawaii Entomol. Soc..

[14] Kuschel G., Grandcolas P. (2008). Curculionoidea (weevils) of New Caledonia and Vanuatu: Basal families and some Curculionidae. Zoologia Neocaledonica, 6. Biodiversity Studies in New Caledonia.

[15] Swezey O.H. (1919). Notes and Exhibitions. Proc. Hawaii Entomol. Soc..

[16] Zimmerman E.C. (1938). On *Chaenosternum* with a key to the genera of Hawaiian Cryptorhynchinae (Coleoptera, Curculionidae). Proc. Hawaii Entomol. Soc..

[17] QGIS Development Team QGIS Geographic Information System. Open Source Geospatial Foundation Projects. http://qgis.org.

[18] Symbiota Collection of Arthropods Network (SCAN): A Data Portal Built to Visualize, Manipulate, and Export Species Occurrences. https://scan-bugs.org/portal/.

[19] Robert V., Stegehuis G., Stalpers J. (2005). The MycoBank Engine and Related Databases. http://www.mycobank.org.

[20] Chamoro M.L., Huang C.L. (2019). Descriptions of the immature stages of *Poteriophorus* Schoenherr, 1838 (Coleoptera: Curculionidae: Dryophthorinae): Larva, pupa, and biology of *Poteriophorus uhlemanni* (Schultze, 1922) discovered through Dawu traditional ecological knowledge. Coleopt. Soc..

[21] Vaurie P. (1967). A revision of the Neotropical genus *Metamasius* (Coleoptera, Curculionidae, Rhynchophorinae). Bull. Am. Mus. Nat. Hist..

[22] Zimmerman E.C. (1968). Rhynchophorinae of southeastern Polynesia (Coleoptera: Curculionidae). Pac. Insects.

[23] *Elytroteinus subtruncatus* (Fairmaire). www.extento.hawaii.edu/kbase/crop/Typeelytrote.htm.

[24] USDA APHIS Regulated Pest List. https://www.invasive.org/species/list.cfm?id=4.

